# SaferBirths bundle of care protocol: a stepped-wedge cluster implementation project in 30 public health-facilities in five regions, Tanzania

**DOI:** 10.1186/s12913-021-07145-1

**Published:** 2021-10-18

**Authors:** Benjamin A. Kamala, Hege L. Ersdal, Estomih Mduma, Robert Moshiro, Sakina Girnary, Ole Terje Østrem, Jørgen Linde, Ingvild Dalen, Elsa Søyland, Dunstan R. Bishanga, Felix Ambrose Bundala, Ahmad M. Makuwani, Boniphace Marwa Richard, Pius David Muzzazzi, Ivony Kamala, Paschal F. Mdoe

**Affiliations:** 1 Department of Research, Haydom Lutheran Hospital, Haydom, Manyara Tanzania; 2 School of Public Health and Social Sciences, Muhimbili University of Health and Allied Sciences (MUHAS), Dar es Salaam, Tanzania; 3 Critical Care and Anaesthesiology Research Group, Stavanger University Hospital, Stavanger, Norway; 4 Faculty of Health Sciences, University of Stavanger, Stavanger, Norway; 5 Department of Pediatrics, Muhimbili National Hospital, Dar es Salaam, Tanzania; 6 Paediatric Association of Tanzania, Dar es Salaam, Tanzania; 7 Laerdal Global Health, Stavanger, Norway; 8 Obstetric Department, Stavanger University Hospital, Stavanger, Norway; 9 SAFER, Stavanger, Norway; 10 Reproductive and Child Health Section, Ministry of Health Community Development, Gender, Elderly and Children, Dodoma, Tanzania; 11 Department of Health, President’s Office- Regional Authority and Local Government, Dodoma, Tanzania; 12 Tanzania Midwifery Association (TAMA), Dar es Salaam, Tanzania

**Keywords:** Safer births, Newborn resuscitation, Helping babies Breathe, Perinatal mortality, Maternal mortality, Stepped-wedge cluster randomization, Moyo, NeoBeat, MamaNatalie and NeoNatalie live

## Abstract

**Background:**

The burden of stillbirth, neonatal and maternal deaths are unacceptably high in low- and middle-income countries, especially around the time of birth. There are scarce resources and/or support implementation of evidence-based training programs. SaferBirths Bundle of Care is a well-proven package of innovative tools coupled with data-driven on-the-job training aimed at reducing perinatal and maternal deaths. The aim of this project is to determine the effect of scaling up the bundle on improving quality of intrapartum care and perinatal survival.

**Methods:**

The project will follow a stepped-wedge cluster implementation design with well-established infrastructures for data collection, management, and analysis in 30 public health facilities in regions in Tanzania. Healthcare workers from selected health facilities will be trained in basic neonatal resuscitation, essential newborn care and essential maternal care. Foetal heart rate monitors (Moyo), neonatal heart rate monitors (NeoBeat) and skills trainers (NeoNatalie Live) will be introduced in the health facilities to facilitate timely identification of foetal distress during labour and improve neonatal resuscitation, respectively. Heart rate signal-data will be automatically collected by Moyo and NeoBeat, and newborn resuscitation training by NeoNatalie Live.

Given an average of 4000 baby-mother pairs per year per health facility giving an estimate of 240,000 baby-mother pairs for a 2-years duration, 25% reduction in perinatal mortality at a two-sided significance level of 5%, intracluster correlation coefficient (ICC) to be 0.0013, the study power stands at 0.99.

**Discussion:**

Previous reports from small-scale Safer Births Bundle implementation studies show satisfactory uptake of interventions with significant improvements in quality of care and lives saved. Better equipped and trained birth attendants are more confident and skilled in providing care. Additionally, local data-driven feedback has shown to drive continuous quality of care improvement initiatives, which is essential to increase perinatal and maternal survival. Strengths of this research project include integration of innovative tools with existing national guidelines, local data-driven decision-making and training. Limitations include the stepwise cluster implementation design that may lead to contamination of the intervention, and/or inability to address the shortage of healthcare workers and medical supplies beyond the project scope.

**Trial registration:**

Name of Trial Registry: ISRCTN Registry.

Trial registration number: ISRCTN30541755.

Date of Registration: 12/10/2020.

Type of registration: Prospectively Registered.

## Background

In 2019, an estimated 1.9 million babies were stillborn at 28 weeks of pregnancy or later, resulting in a global stillbirth rate of 13.9 per 1000 total births [1]. Additionally, there were 2.4 million neonatal deaths in 2019 – approximately 6700 neonatal deaths every day – with about a third of all neonatal deaths occurring within the first day after birth, and close to three-quarters occurring within the first week of life [2]. The global maternal mortality has declined by around 38% between 2000 to 2017, and currently the rate stands at about 211 deaths per 100,000 live births [3]. The low-and-middle income countries (LMICs) contribute significantly (approximately 98%) to global rates of stillbirths andneonatal and maternal mortality. Sub-Saharan Africa contributes to about 50% of these deaths, mainly due to a major gap between healthcare demand and provision of services [4].

In 2016, Tanzania recorded 20 stillbirths/1000 births and 25 neonatal deaths/1000 live births. Most of these deaths were associated with challenges in providing care around the time of birth [4–6]. To achieve the Sustainable Development Goal (SDG) target 3.2 of less than 12 deaths per 1000 live births by year 2030, Tanzania must halve neonatal mortality within the remaining 10-year period. Additionally, rates of maternal mortality are also very high. Tanzania reported 556 maternal deaths/100,000 live births in 2019, translating to 1 in 33 women dying in relation to pregnancy and childbirth [7], mostly due to postpartum haemorrhage (PPH).

Causes of stillbirths and neonatal deaths are intricately linked. They are usually obstetric in origin and strongly associated with the causes of maternal mortality and morbidity [8]. As a result, factors associated with perinatal deaths may be an indirect measure of the availability and quality of care provided during childbirth and the neonatal period [9, 10]. Dying on the day of birth is still commonplace around the world, particularly in LMICs [11–15], indicating inadequate quality of care around labour, delivery and immediate neonatal period. Both perinatal deaths (i.e. intrapartum related/fresh stillbirths (FSB) and neonatal deaths) and maternal deaths can be substantially reduced by improving quality of care around time of labour and birth [16].

Inadequate foetal heart rate (FHR) monitoring during labour, substandard partogram use and obstetric complications including obstructed/prolonged labour, preeclampsia/eclampsia and antepartum haemorrhage are among the identified factors of perinatal mortality and morbidity [17, 18]. Most intrapartum related deaths could be prevented through better FHR monitoring, coupled with timely obstetric actions [19–23]. FHR monitoring using Moyo has shown to improve quality of labour management by increasing the frequency of FHR assessments, timely identification of abnormal FHR, increased frequency of intrauterine resuscitation, and improved partogram use [24–26].

Around 10–15% of live births need basic resuscitation such as stimulation, and 5–8% require positive pressure ventilation (PPV) to initiate breathing [22, 23, 27]. Provision of adequate PPV after birth is crucial to avoid complications related to birth asphyxia [23, 28]. Most asphyxia-related fatalities are preventable with low-cost, basic, but prompt care at the time of birth [29] [19–21]. Newborns with heart rate activity are sometimes misclassified as fresh stillbirths (FSB) [20]. Timely identification of non-breathing newborns with heart activities immediately after birth, coupled with timely and effective PPV, can significantly reduce misclassification of FSB, decrease asphyxia-related deaths and prevent long-term impairment [11, 16–18].

The Helping Babies Breathe (HBB) study conducted in 8 referral health-facilities in Tanzania from 2010 to 2011 showed a remarkable 47% reduction in all-cause newborn mortality within the first 24 h of life and another 24% reduction in FSB [30]. However, the “high skills” attained by 87% of healthcare providers when tested immediately after training declined to 56% when tested 4–6 months post-training [31]. It is further reported that a one-day HBB training, as conducted in Tanzania, did not facilitate translation of acquired skills into improved clinical management [32]. However, implementation of frequent and brief onsite HBB training, supplemented with feedback and supportive supervision, did effectively improve clinical care and perinatal outcomes [33]. Furthermore, it has been suggested in a HBB Delphi review that training of clinical staff should be augmented with simulation methods to build confidence and competence among healthcare workers [34], and be linked with clinical debriefings [35] to increase perinatal survival globally. The facility birth rate has increased steadily in Tanzania, from less than 50% in 2010 to 63% in 2017 [4]. However, this increase does not similarly reflect a decline in perinatal and maternal mortality. Increase in facility births need to be coupled with quality of care provision by competent and better equipped healthcare workers in order to reduce both perinatal deaths and maternal morbidity and/or mortality [36–38]. A recent report showed that more deaths, including neonatal deaths, occur due to inadequate quality care rather than underutilization of care [39]. Incentivizing facility delivery, where competent trained and equipped healthcare workers are able to safeguard uncomplicated births and provide good quality emergency obstetric and newborn care, is likely to improve perinatal and maternal outcomes [25, 27].

Through the Safer Births project (www.saferbirths.com) several innovations and interventions have been studied separately and proven feasible and effective in both urban and rural low-resource settings [35, 40–44] . The SaferBirths Bundle of Care (SBBC) is a package combining these proven innovative clinical tools (Moyo, NeoBeat and Upright bag mask) and training tools (MamaNatalie and NeoNatalie Live) with an on-the-job low-dose, high-frequency (LDHF) simulation-based training model, utilizing local data and feedback loops [33]. Reinforcing competencies through regular onsite LDHF simulation-based training with feedback, and providing targeted training based on clinical data that highlights areas in need of improvement, can motivate and guide healthcare workers on how to improve clinical care [33]. We hypothesize that this training model will increase knowledge and skills, help translate what is learned into improved clinical practice and establish a culture of excellence within the facilities over time. Furthermore, we hypothesize that these improvements, together with the innovative clinical tools, will halve perinatal mortality over a three-year period.

### Aim of the project

The aim of this project is to describe the SBBC implementation process and determine the impact on care provision and perinatal and maternal outcomes after scaling up SBBC to 30 selected health facilities in 5 regions of mainland Tanzania. Specifically, the project aims to;

1. Document implementation of the training cascade and systematic LDHF onsite simulation training and assess the effectiveness on skills acquisition and retention over time
2. Document changes in quality of care over time
3. Document changes in perinatal and maternal outcomes over time
4. Explore healthcare workers’ experiences of providing care after implementation of SBBC in their facilities
5. Explore parturient women’s experiences of the SBBC to identify potential areas of improvement
6. Identify enablers and barriers as reported by key stakeholders, for further scale-up of SBBC

## Methods

### Study design

**Fig. 1:**
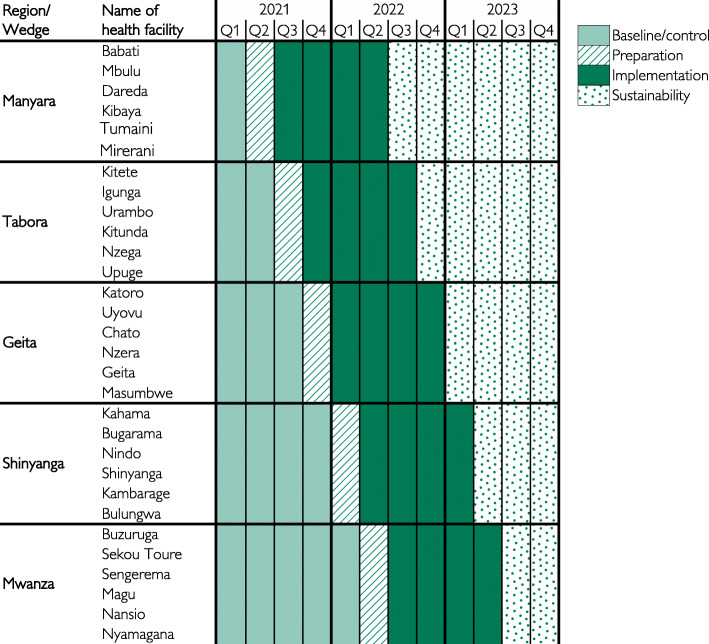
Stepped-wedge cluster randomized trial in 30 health facilities in 5 regions

This is a continuous quality improvement (CQI) project, which will be implemented in a stratified stepped-wedge cluster (regions) randomisation approach (Fig. 1). Randomization was done using simple random sampling i.e. the first region, Manyara, was purposively selected for logistical and strategic reasons, whereas subsequent regions were selected randomly. Clusters will receive the intervention at different time points based on the randomization [45]. Prospective observational data will be collected before and after introduction of the intervention periods at each cluster. The before implementation data, collected for three to 12 months (Fig. 1), will serve as baseline/control data. Random and sequential crossover of clusters from control to intervention will be conducted until all clusters are exposed. More clusters will be exposed to the intervention towards the end of the study.

#### Q stands for quarter of the respective year

This design is preferred because, firstly, it provides a robust scientific evaluation of health service delivery of interventions, and it does not involve individual patient recruitment, rather a cluster (i.e. health facilities in one region). Secondly, for ethical purposes it ensures that all clusters receive the proven beneficial interventions by the end of the project. Thirdly, not all health facilities will implement the bundle at the same time, but in a stepwise manner, providing a learning opportunity for more optimal implementation at the next facilities. Data collected at each facility and context-specific issues will inform local training models and use of effective plan-do-study-act (P-D-S-A) cycles as study implementation continues [46, 47].

### Sites

The project will be implemented in 30 health facilities in 5 clusters (regions) in mainland Tanzania: Manyara, Tabora, Geita, Shinyanga, and Mwanza as described in Fig. 1. These health facilities were selected in consultation with the Tanzania Ministry of Health Community Development, Gender, Elderly and Children (MoHCDGEC) and the President’s Office- Regional Administration and Local Government (PO-RALG) based on the following criteria: 1) high burden of maternal and perinatal mortality, 2) high volume of deliveries, and 3) alignment with the ministry’s strategic priorities for maternal and newborn interventions in the country. Table 1 shows total births, fresh stillbirth rates, macerated stillbirth rates, 7-days neonatal mortality and maternal mortality rates in 2019 for the 30 health facilities. The data was obtained from respective health facility records of the health management information system. All of the health facilities provide Comprehensive Emergency Obstetric and Basic Newborn Care services and have separate labour units and operating theatres.

**Table 1:** Total birth and mortality rate in the study hospitals (2019)

Region	Health Facility	Births	Fresh Stillbirth rate (per 1000 live births)	Macerated Stillbirth rate (per 1000 births)	7-day neonatal mortality rate (per 1000 live births	Maternal mortality per 100,000 births
Manyara	Dareda	3333	2.4	14.1	6.0	NA
	Babati DH	3342	1.5	5.7	3.0	149.6
	Tumaini DH	2964	6.1	6.1	4.0	NA
	Kibaya DH	1808	21.6	16.6	13.3	165.9
	Mbulu DH	3275	6.4	6.4	NA	NA
	Mererani HC	1123	0.9	5.3	NA	NA
Tabora	Igunga DH	5378	11.2	11.5	10.6	185.9
	Nzega DH	4441	7.4	18.7	NA	NA
	Kitunda HC	1331	6.0	6.0	Na	NA
	Kitete RRH	5202	18.5	22.9	4.6	NA
	Urambo DH	2812	26.3	19.2	1.4	106.7
	Upuge HC	579	15.5	NA	NA	NA
Geita	Uyovu HC	3945	0.5	1.8	1.0	50.7
	Chato DH	3430	2.3	1.5	2.3	145.8
	Katoro HC	7154	3.5	3.2	0.3	83.9
	Nzera DH	3974	1.5	1.5	NA	75.5
	Geita RRH	7199	18.9	16.8	13.3	291.7
	Masumbwe HC	3296	2.1	3.6	NA	364.1
Shinyanga	Kahama DH	9559	14.8	17.9	10.5	73.2
	Bugarama HC	1757	1.1	0.6	NA	NA
	NINDO HC	1993	8.5	5.0	4.5	351.2
	Kambarage HC	2512	4.8	3.2	NA	NA
	Shinyanga RRH	4206	30.9	20.4	25.9	428.0
	Bulungwa	1951	0.5	NA	NA	NA
Mwanza	Buzuruga HC	2579	0.8	5.8	1.6	NA
	Sekou Toure RRH	9024	15.1	19.8	6.6	110.8
	Magu DH	3304	13.3	21.5	NA	NA
	Nyamagana DH	7619	7.9	11.8	1.4	78.8
	Sengerema DDH	10,064	10.2	12.2	19.7	218.6
	Nansio DH	4339	11.5	7.1	NA	NA

*NA* Data was not available for that particular health facility, *DH* District Hospital, *RRH* Regional Referral Hospital, *HC* Health Centre

### Description of the SaferBirths bundle of Care (SBBC) interventions

**Fig. 2:**
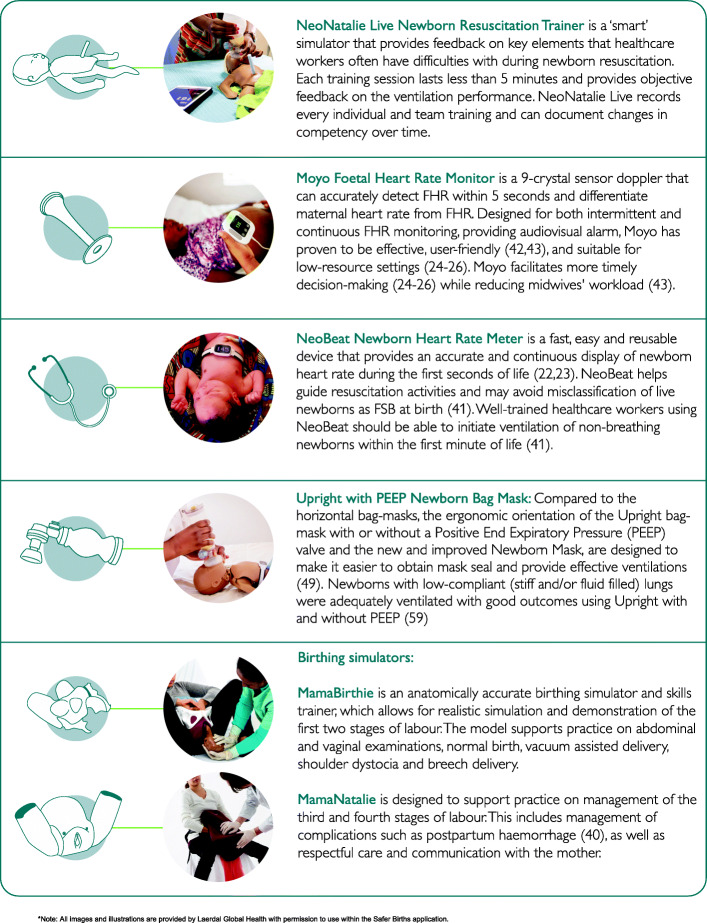
Description of the innovative tools in the SaferBirths Bundle of Care package (Laerdal Global Health, Stavanger, Norway)

The SBBC is a quality improvement combination of innovative clinical and training tools (Fig. 2) coupled with LDHF on-the-job training aimed at empowering healthcare workers to improve care around labour and delivery.

**Fig. 3:**
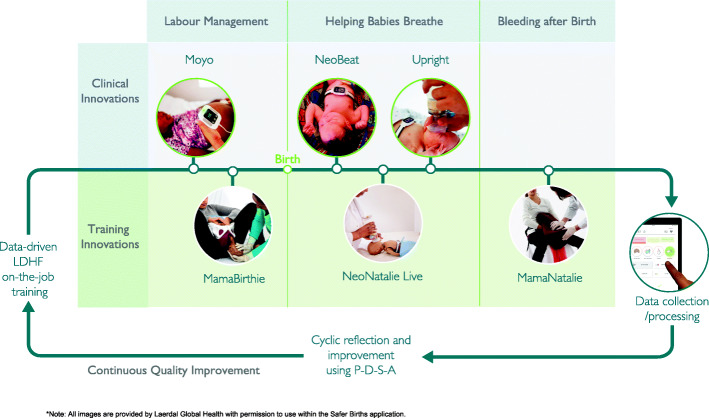
Interventions that will be implemented during the SaferBirths Bundle project period

The interventions will target three main areas: labour management, newborn resuscitation (HBB), and management of bleeding after birth (Fig. 2). SBBC will provide a combination of training and clinical solutions for these situations including a data management system that provides rapid local feedback (Fig. 3). The bundle is the result of 10 years of a multidisciplinary collaboration between international institutions from within and outside Tanzania. All the innovative tools were co-created with midwives and doctors working in maternity departments at Haydom Lutheran Facility (rural setting), Muhimbili National Facility (urban), Temeke Referral Regional Hospital (urban), researchers from Stavanger University Hospital/SAFER and engineers from Laerdal Global Health (saferbirths.com/publications).

**Fig. 4:**
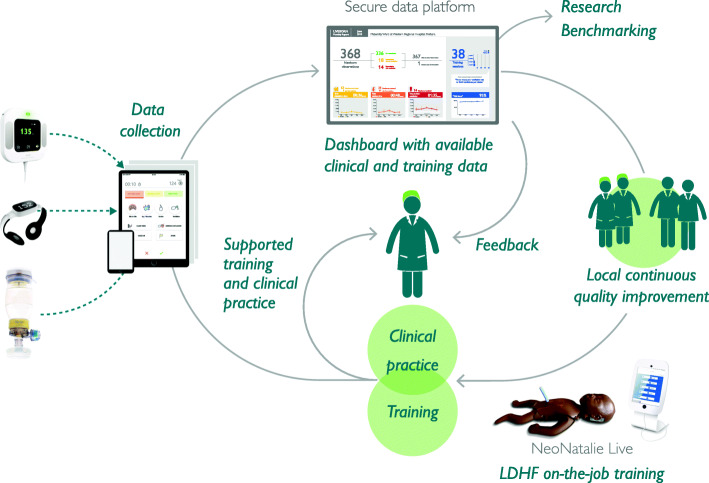
Integration of innovations, data capturing and feedback mechanisms

The SBBC interventions will be coupled with semi-automatic data capturing components, whereby clinical data through the use of Moyo, NeoBeat and the Liveborn App, and training data through the use ofNeoNatalie Live will be uploaded to a secure access-controlled research server. Data will be automatically processed, and basic statistics andanalyses will be easily available on local dashboards (Fig. 3). This data can be shared with local healthcare workers, used to facilitate PDSA cycles (Fig. 2) and for benchmarking between facilities. Such availability of data will enable a system with rapid and objective feedback on training, clinical quality of care, and patient outcomes (Figs. 4) to guide new efforts (PDSA-cycles) and potentially increase motivation for CQI. Specifically, each health facility will utilise the objective data-driven feedback to adjust ongoing LDHF on-site simulation training, targeting the identified gaps in clinical care.

### Implementation strategy

**Fig. 5:**
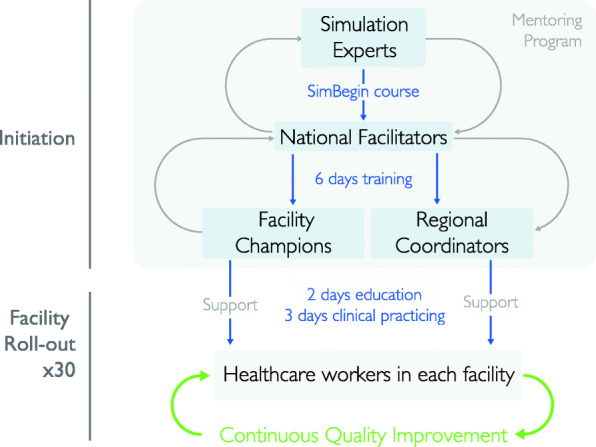
Illustration of the planned training cascade and implementation strategy

SBBC is coordinated by Haydom Lutheran Hospital in collaboration with the two Tanzanian sectoral Ministries in Health (MoHCDGEC and PO-RALG), the Tanzanian Midwifery Association (TAMA) and the Paediatric Associations of Tanzania (PAT) to scale up and ensure that implementation is rooted in the local health organizations. Project investigators, in collaboration with experts from SAFER (a simulation and implementation center based in Stavanger, Norway), will conduct courses in newborn care, labour management and a basic simulation-based train-the-trainer course (SimBegin) of 15 selected national candidates from TAMA and PAT. These 15 participants will become national facilitators and conduct cascade trainings of facility champions on newborn care, labour management and simulation-based training in the five regions. Figure 5 describes the training cascade that will be implemented. Building this capacity within the professional associations means the value can be applied not only to the SBBC project, but also to other training initiatives around the country where simulation-based learning andragogy can be useful.

#### HCW = healthcare workers

Two facility champions from each facility will be trained to become site facilitators (Fig. 4). In total, there will be 60 facility champions (2 × 30 facilities) trained by the national facilitators for 6 days in 5 batches (stepwise for each region) with focus on 1) the use of the innovative tools, 2) how to facilitate simulation based LDHF on-the-job trainings, and 3) collection and use of local data for rapid feedback and PDSA CQI. Regional coordinators (*n* = 5) will be oriented on the SBBC interventions and how to coordinate technical and administrative issues in each region. Standardised methods will be used to ensure that participants are sufficiently trained, including those outlined in WHO Standards for Improving Quality of Maternal and Newborn Care in health facilities [48].

Following the six-day training of facility champions, a five-day training will be conducted at each facility, targeting all healthcare workers and service providers at the facility maternity and newborn care units. The training will be divided into a theoretical educational part (2 days) and a clinical practicing part including simulations (3 days) (Fig. 4). The two facility champions at each site will assist 2 national facilitators to conduct these trainings.

The 30 health facilities will be provided the innovative training, clinical and data management tools, which will remain at the facility beyond the project implementation period to sustain improvement efforts. With support from the project’s regional coordinators, the facility champions will be responsible for following up day-to-day duties in relation to the bundle and facilitating LDHF on-the-job trainings related to newborn resuscitation and management of various labour complications. Additionally, the facility champions will utilize the captured data to conduct scheduled debrief meetings to enable healthcare workers to reflect on the care they provide. To ensure that facility champions are well-supported in their new role, national facilitators, in collaboration with the regional coordinators, will conduct scheduled supportive supervision to provide in-house training and support on a regular basis. A mentorship program will be developed and implemented in close collaboration with simulation experts at SAFER to support especially the national facilitators (faculty development) (Fig. 4).

Prior to introduction of the SBBC interventions at the facility level, situational analysis meetings will be conducted with clinical staff at the different sites in consultation with regional, council and facility health management teams. The aim will be to assess and identify bottlenecks related to readiness, availability, and quality of provided intrapartum care. The results of these assessments will be used to cater implementation of the bundle to meetthe needs of the respective health facilities.

### Outcomes

#### Primary outcome

- Perinatal mortality defined as FSB (i.e. stillborn baby with no signs of life at delivery and ≥ 28 weeks of gestation with intact skin and no signs of disintegration in utero) and neonatal death within the first 24 h of life [49]

#### Secondary outcomes

- Proportion of deliveries with FHR monitoring as per standard protocol
- Proportion of deliveries resulting in emergency caesarean sections or instrumental deliveries
- Proportion of deliveries where abnormal FHR detection is followed by neonatal resuscitation
- Proportion of neonates who receive PPV
- Proportion of neonatal morbidities (i.e., resuscitation, low Apgar score, and admission to neonatal units within 24 h after birth)
- Proportion of mothers with postpartum haemorrhage managed successfully
- Maternal mortality
- Proportion of healthcare workers maintaining neonatal resuscitation skills 6 months, 1, 2 and 3 years after implementation of LDHF training and targeted PDSA cycles
- Healthcare workers’ attitudes, perceptions and acceptability towards the SBBC
- Parturient women’s experiences and perceptions about the SBBC
- Key stakeholders’ experiences and perceptions about enablers and barriers for efficient scale up of SBBC

### Study population and sample size estimation

The study will include parturient women, their offspring, and healthcare workers in the 30 selected health facilities. Pregnant women at gestational age 28 weeks and above with a live foetus at labour admission will be enrolled in the study. All healthcare workers in the maternity ward (prenatal, labour, postnatal and neonatal wards) in the selected health facilities will be involved.

We used Stata function stepped-wedge to calculate the power [50] with the following input parameters. An average of 4000 baby-mother pairs per year per health facility giving an estimate of 240,000 baby-mother pairs for a 2-years duration (i.e., an average of 1 year pre-implementation and 1 year post implementation), 25% reduction in perinatal mortality from 2 to 1.5% at a two-sided significance level of 5%. Assuming intracluster correlation coefficient (ICC) to be 0.0013 [51], the study power stands at 0.99.

Purposive sampling will be used in the qualitative components of the study. Maximum variation sampling will guide the selection of participants for in-depth interviews and focus group discussion with healthcare workers, facility management team as well as mothers receiving care. This approach secures a wide variety of people of interest and consequently a broad range of perspectives to better understand contextual factors influencing implementation. Qualitative data will be collected until saturation is achieved.

### Data management

#### Data collection

Research assistants (at least two at each site) dedicated for data collection and regional coordinators (one for each region) will be trained on data collection and quality control procedures, including correction of errors and safe transfer of data to the central research server. The clinical data (process and patient outcomes) collected at each facility will be entered into an electronic data collection system i.e. Open Data Kit (ODK) developed specifically for this project. Refresher training of research assistants will be conducted by the study investigators and data manager as needed. Data will prospectively be collected at all sites for a period of 3 years. Clinical process and patient data will not include personal information: instead a unique identification (ID) number will be provided. The regional coordinators will oversee the data collection processes at the regional level. Several datasets will be collected during both baseline and implementation phases. We will monitor implementation level data (e.g. facilitators and barriers for scale-up), process level data (e.g. the adoption of training, use and experiences with the SBBC tools), and outcome/output level data (patient outcomes, clinical actions, and biomedical signal data).

##### Implementation level data

A facility readiness and service availability assessment tool will be used to conduct bottleneck analyses of each health facility readiness for good quality intrapartum care. A quality improvement tool will be used to develop a specific plan based on the bottleneck analysis. Knowledge about CQI process implementation at SBBC sites will be collected using Logbooks and PDSA diaries.

##### Process level data

Systems will be established to document the dissemination of trainings and the frequency and quality (performance) of training among healthcare workers in each site over the 3 years. On-site simulation training data will be semi-automatically collected by the NeoNatalie Live simulator and uploaded automatically to the central server at Haydom. Healthcare workers’ knowledge and skills on intrapartum care before and after the initial 5-day training will be collected using multiple-choice questions and OSCEs. Their periodic skills retention tests will be done using OSCEs.

The adoption of the SBBC will be further evaluated through in-depth interviews and focus group discussions with healthcare workers, caregivers, and facility leaders/management. Healthcare workers will be asked to ascertain acceptability and barriers of the new interventions. The perceptions of women about intrapartum care will be assessed with semi-structured interviews.

##### Outcome/output level data

Patient morbidity, mortality and clinical events/actions during intrapartum care data will be collected from patient case notes, labour and delivery registers. The use of clinical SBBC tools (Moyo, NeoBeat and Upright bag mask) will be recorded on separate data collection forms Biomedical heart rate signal data will be automatically collected by Moyo (foetal heart rate) and NeoBeat (newborn heart rate).

#### Data transfer and quality control

In each facility, the research assistants will assess the quality and completeness of the data before uploading via the electronic data collection system into the central secure research server/database at Haydom Lutheran Hospital. Signal data collected by Moyo and NeoBeat will automatically upload to the research server. The regional coordinators will perform quality data check on a daily basis. A data manager, located centrally at Haydom, will be responsible for overseeing the data management process including quality control. The data manager will run queries and perform final quality control before saving the data on the secure research server. Any querry identified will be sent back to the regional coordinator who will communicate with the research assistants for resolution. Training data will be collected partly automatically by the NeoNatalie Live simulator and others will be manually entered into the database immediately after training. De-identified qualitative data will be collected using digital recorders. They will be transcript, processed and stored in password protected research computers with controlled access.

#### Feedback of data

After data correction and cleaning, the data manager will prepare summary tables of selected variables identified for rapid evaluation of study progress. The summary tables will be shared with the regional coordinators, health management team in the regions and health facilities, and the national and site facilitators. The feedback will then be shared with healthcare workers and utilized to assess the strengths and identify procedure-practice gaps which need improvement, in line with the PDSA model and CQI. Some facilities will have direct access to their own “dashboard” (Fig. 3) and can follow their daily statistics.

### Data analysis

A final data analysis plan will be established in collaboration with the respective ministries, principal investigators and statistical partners in the consortium to answer all the specific project objectives. For binary correlated data (e.g. before and after training practices for health care workers), the one-sided McNemar’s test will be considered. Differences in training attendance and knowledge and skills acquisition rates between health-facilities will be compared using both bivariate and logistic regressions. The training scores will be modelled as longitudinal data and compared over time using time series analyses and mixed model effects. Statistical process control methods will also be utilised. The clinical care indicators and perinatal/maternal outcomes will be analysed and compared both within and between clusters/facilities (and regions) by random intercept models factoring in the stepped-wedge design. Poisson regression models will be used to estimate the differences in rates of morbidity and mortality. Qualitative thematic content data analysis methods will be used to analyse qualitative data [52]. Qualitative data will be processed and analysed using COREQ guidelines [53].

## Discussion

Successful implementation of SBBC in Tanzania has the potential to improve quality of care and save thousands of lives during labour and after birth. Better trained and equipped healthcare workers in health facilities with access to LDHF training, and data-driven feedback stand a better chance to save more perinatal and maternal lives.

The SBBC will provide healthcare workers with Moyo FHR monitors to facilitate labour management, i.e. timely identification of foetuses who needs urgent obstetric interventions. The use of NeoBeat for quick assessment of non-breathing neonates immediately after birth will help distinguishing true FSB from severely asphyxiated neonates, reducing misclassification of FSB and facilitate timely decisions to start resuscitation. The use of training equipment (MamaNatalie and NeoNatalie) including CQI PDSA cycles, will ensure skills promotion, retention and build competence and confidence among the healthcare workers.

Previous Safer Births studies [23, 28, 35, 43, 44, 54–58] testing the individual innovations and/or interventions on a smaller scale, have reported improvements in quality of care and saving lives. Generating evidence-based knowledge on quality of care around birth from large scale interventions is a key priority to reach the SDG targets 3.1 and 3.2 of reducing preventable maternal and perinatal mortalities [59, 60]. Facilities need to provide a safe and professional environment, enabling well-educated and skilled healthcare workers to perform optimally with essential resources available (SDGs 3c/4.7) [61, 62]. This is simultaneously pivotal for assuring respectful working conditions for healthcare workers (SDG 8.8).

The Safer Births innovations (www.saferbirths.com) have been developed, tested separately, and proven effective in changing clinical behaviour and improving birth outcomes to cater for these global SDG targets. We will now study the impact of the combined SBBC package on improving quality of intrapartum care and perinatal outcomes at scale. In addition, the project will provide contextual information on requirements to scale up implementation of these innovative interventions to health-facilities in low resource settings.

There are several strengths of our study. Firstly, we will integrate the new clinical and training SBBC tools with existing national guidelines and initiatives to ensure sustainability of the interventions. Secondly, we will use the proven LDHF on-the-job training concept, utilizing birth and newborn simulators, to ensure skills acquisition, translation, and retention. Thirdly, the study will involve rapid local data-driven feedback to stimulate CQI efforts, improving quality of care and patient outcomes at the local facility.

The project has several limitations especially related to the stepped-wedged design. Firstly, this design includes a potential contamination risk of the baseline/control periods of later wedges/clusters, which may dilute the effects. Secondly, unlike the traditional cluster randomised trials, the stepped-wedge design may pose a challenge of adherence to the randomisation sequence as some regions may feel they are being delayed in implementing the interventions. Thirdly, if national policy changes occur, they may affect some of the wedges/clusters and accommodating these changes during the trial period pose a challenge [63]. Fourthly, in the SBBC project, a number of clusters (hospitals) will be allocated to a number of wedges (regions). Each wedge will contain a relatively small number of clusters (*n* = 6) receiving the intervention at different times. The final large number of clusters (*n* = 30) and the various crossovers taking place at different times, make it difficult to adapt the CONSORT diagram, often used when reporting classical cluster randomised controlled studies [64]. Another feature posing challenges in reporting results, is the assessment of whether the randomisation has resulted in balanced study conditions at baseline given the small number of clusters in each wedge. For this reason, a baseline table will be used to present baseline characteristics of all clusters [64, 65].

Our findings will be published as multiple papers in peer-reviewed relevant Open-Access international journals and communicated on global conferences. Summary reports, in popular language, will be developed for policymakers; outlining required quality improvement frameworks for improving care to advocate for more investments in such interventions. Combined workshops and dissemination seminars with relevant stakeholders will be conducted locally. The aim will be to summarize and discuss the findings from the ongoing data collection and related implications for operational changes in care at birth, as well as in development and implementation of PDSA cycles as part of CQI.

## Data Availability

This is a study protocol and data are not available at this stage. After data collection is completed, a de-identified dataset may be made available upon reasonable request of the corresponding author once the study is complete.

## Abbreviations

LMIC: Low and Middle Income Countries
FSB: Fresh Stillbirths
SDG: Sustainable Development Goal
PPV: Positive Pressure Ventilation
PPH: Post-Partum Haemorrhage
LDHF: Low-Dose-High-Frequency
PO-RALG: Presidents Office: Regional Authority and Local Government
MOHCDGEC: Ministry of Health, Community Development, Gender, Elderly and Children
CQI: Continuous Quality Improvement
PAT: Paediatric Association of Tanzania
TAMA: Tanzania Midwifery Association
OSCE: Objective Structured Clinical Examination
COREQ: Consolidated Criteria for Reporting Qualitative Research
HBS: Helping Babies Survive
MDG: Millennium Development Goal
PDSA: Plan-Do-Study-Act
ANC: Antenatal care
STROBE: The Strengthening the Reporting of Observational Studies in Epidemiology
DE: Design Effect
ICC: Intra-Cluster Correlation Coefficient
IDI: In-Depth Interviews
FGD: Focus Group Discussion
